# Test-retest of computerized health status questionnaires frequently used in the monitoring of knee osteoarthritis: a randomized crossover trial

**DOI:** 10.1186/1471-2474-12-190

**Published:** 2011-08-18

**Authors:** Henrik Gudbergsen, Else M Bartels, Peter Krusager, Eva E Wæhrens, Robin Christensen, Bente Danneskiold-Samsøe, Henning Bliddal

**Affiliations:** 1 The Parker Institute, Frederiksberg Hospital, Nordre Fasanvej 57, 2000 Frederiksberg, Copenhagen, Denmark; 2 Institute for Sports and Biomechanics, University of Southern Denmark, Campusvej 55, 5230, Odense M, Denmark; 3 Faculty of Health Science, University of Copenhagen, Blegdamsvej 3b, 2200, Copenhagen NV, Denmark; 4 Centre for Sensory-Motor Interaction, Aalborg University, Fredrik Bajers Vej 7 D3, 9220, Aalborg, Denmark

## Abstract

**Background:**

To compare data based on touch screen to data based on traditional paper versions of questionnaires frequently used to examine patient reported outcomes in knee osteoarthritis patients and to examine the impact of patient characteristics on this comparison

**Methods:**

Participants were recruited from an ongoing trial (http://ClinicalTrials.Gov Identifier: NCT00655941). 20 female participants, mean age 67 (SD 7), completed KOOS, VAS pain, function and patient global, SF-36, Physical Activity Scale, painDETECT, and the ADL Taxonomy. Patients were randomly assigned to one of two subgroups, completing either the paper or touch screen version first. Mean, mean differences (95% CI), median, median differences and Intraclass Correlation Coefficients (ICCs) were calculated for all questionnaires.

**Results:**

ICCs between data based on computerized and paper versions ranged from 0.86 to 0.99. Analysis revealed a statistically significant difference between versions of the ADL Taxonomy, but not for the remaining questionnaires. Age, computer experience or education-level had no significant impact on the results. The computerized questionnaires were reported to be easier to use.

**Conclusion:**

The computerized questionnaires gave comparable results to answers given on paper. Patient characteristics did not influence results and implementation was feasible.

## Background

In the Rheumatology clinic, self-administered Health Status Questionnaires (HSQs) are an important part of the overall evaluation of patients [1,2]. Several questionnaires are applied in the self-assessment process, and data handling can be tedious, expensive and open to errors, when data are being transferred manually from paper into electronic systems. Implementation of computerized methods of data collection based on touch-screen would be more cost-effective and decrease the risk of error.

Touch screen is a new tool applied in places like libraries and shops, as well as in health care settings [3-5]. However, prior to implementation of data collection based on touch screen, it is crucial to evaluate if questionnaires based on paper and touch screen provide similar information. Furthermore, as clinical studies often include several questionnaires it is relevant to validate multiple computerized questionnaires for the retrieval of patient-reported outcomes (PROs).

Previous studies have examined groups of patients with different diagnoses by a wide variety of computer equipment, but little has been done within the field of knee osteoarthritis (KOA) [1,2,6,7]. As the number of KOA patients are estimated to increase dramatically in the future, there is a huge need for an easy and precise method for retrieval of PROs from this patient category [8,9].

For the single most common arthritic disease, KOA [10], a selection of relevant HSQs are: Knee Osteoarthritis Outcome Score (KOOS) [11], VAS pain, function and patient disability [12], Medical Outcomes Study 36-Item Short-Form Health Survey (SF-36) [13], Physical Activity Scale [14], painDETECT [15] and Activity of Daily Living (ADL) Taxonomy [15]. Transfer of these from paper to touch screen will, apart from making the whole data collection more cost-effective, prevent missing data, avoid problems and errors during data transfer, and possibly make answering the questions easier [15,16].

The aim of our study was to compare data based on touch screen to data based on paper version of the above-mentioned PROs to determine if the two versions are comparable. Secondly, we aimed to examine the impact of patient characteristics on differences between questionnaire-versions. Thirdly, we examined the patients' acceptance of computerized questionnaires.

## Methods

### Participants

Participants were recruited in March and April 2010 from an ongoing in-house KOA trial (the CAROT-study; http://ClinicalTrials.Gov Identifier: NCT00655941) at The Parker Institute, Frederiksberg Hospital, Denmark. Participants from the CAROT-study were consecutively invited to also participate in this study and recruitment ended when a sufficient number of patients were included. The participants were prior to participation informed of the content of the study.

### Study design

**Figure 1:**
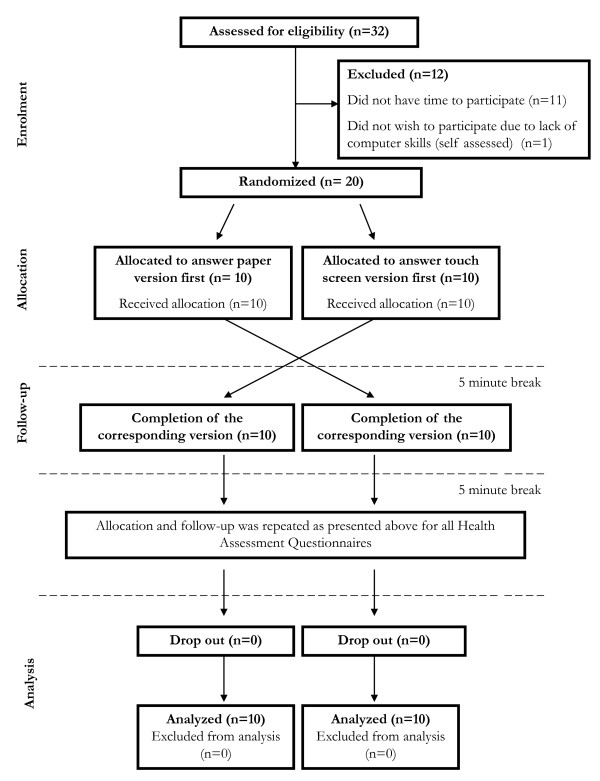
**Flow diagram of study design**.

The study was carried out in a repeated randomized crossover design (Figure 1). That is, patients were randomly assigned to one of two subgroups, completing either the paper or touch screen version first in a waiting-room setting at the hospital (trial profile, see Figure 1). The order of questionnaires was held constant, but patients entered the sequence at different points starting with either the paper or touch screen version. Patients completed both versions (paper and touch screen) of all questionnaires with a 5-minutes interval between versions, and a 5-minutes break between questionnaires. Patients were asked to fill in the paper versions the way they normally would do it. To complete the touch screen versions patients were placed in front of the computer screen and asked to follow the instructions on the screen. No information was given beforehand, but a readily available instructor was present to provide tutoring on demand.

### Data collection

Touch screen data was instantly exported to a specific database, whereas data from paper versions was manually entered into the same database. Time used on each touch screen HSQ was measured in seconds. Staff not involved in the recording session later checked the latter dataset.

In order to assess the feasibility and acceptability of computer-based questionnaires, the participants filled in an additional questionnaire regarding level of education, previous computer experience, and method preferences.

### Questionnaires

Criteria for selecting questionnaires were: relevance to KOA, designed for self-administration, and together representing a wide variety of questionnaire types. Furthermore, these questionnaires were familiar to the participants as they were also applied in the CAROT-study.

The Knee Injury and Osteoarthritis Outcome Score (KOOS) exploit impairment, disability and handicap within 42 items in 5 domains (activities of daily living, pain, knee-related quality of life, symptoms and sport/recreation). Items are scored from 0-4 and then transformed into a 0-100 scale; 0 representing extreme knee-related problems and 100 representing no knee-related problems [11,15].

VAS pain, function and patient global scales (0 to 100 mm), which are used in the OMERACT-OARSI responder criteria; tools used for outcomes assessment in KOA research [12].

The SF-36 questionnaire includes 8 multi item domains (physical function, social function, role-emotional, role-physical, bodily pain, general health, mental health and vitality). These can be combined into 2 summary measures (physical and mental component summary measures). The scales are linearly transformed to a 0 to 100 scale; 0 indicating the least favourable health state and 100 indicating the best state of health [13].

The Physical Activity Scale assess physical activity in metabolic equivalents (METs) as patients report the amount of time (0 to 24 hours) they spend on 9 different levels of activity on an average weekday; in this trial the total amount of time had to reach 24 hours [14].

The painDETECT questionnaire is used to evaluate whether chronic pain patients suffer primarily from nociceptive or neuropathic pain [15]. This questionnaire has several subscales and we analyzed subscales as follows: item 1-3 (10-point-Likert scales), item 4 (four figures of pain symptom variation), item 5 (yes/no to symptoms of radiating pain), item 6 (average value of seven 5-point-Likert scales) as well as a total painDETECT score.

The ADL Taxonomy is an instrument that is used to evaluate the patient's ability to perform 47 personal and instrumental activities of daily living (ADL) [17]. We used the Rasch-based questionnaire version (ADL-Q) [18] and Rasch computer software were employed to convert the ordinal ADL Taxonomy ratings into linear measures of self-reported ADL ability.

### Setup

The computer-assisted questionnaire solution was freeware-based on Microsoft Visual Studio Professional, using Language Integrated Query (modelling), Visual C# (computer language), Microsoft SQL Server Express (database management and maintenance) and GIMP (freeware image managing software). The underlying database was a Microsoft SQL Server database (saved as an mdf file), built up and modified by the use of SQL Server Management Studio (Microsoft^®^). The database was built on a relational database design. The entire solution ran on Windows 7. Requirements were Microsoft .NET Framework 3.5, and in order to ensure optimal image quality, the programme was run on a 19-inch Flex Panel PC Wide - Atom touch screen (16:10 Blue Line A/S, Århus N, Denmark, http://www.blue-line.dk).

The screen was wall-mounted and a single question appeared and was answered by tapping with either a finger or a stylus pen. Patients could freely choose the optimal position of the screen (built-in mobility in 3D), in order to ensure that patients had as much privacy as possible, when replying on the screen..

The patient interface was designed to be user-friendly with large visible characters. The questions were answered by placing a bar on a 100 mm horizontal VAS-scale or by marking the relevant squares on e.g. a Likert scale. In addition to these features, the patient was at any time able to tap on: "forward" and "backward". Each questionnaire had to be fully answered before it was possible to continue.

The platform fulfilled all legal requirements regarding protection of patient-sensitive data.

### Ethics and consent

Patients gave their consent prior to inclusion. According to Danish law this study did not require approval by the Ethics Committee.

### Statistics

Differences were calculated by subtracting scores from each question on paper versions from the corresponding scores on touch screen versions.

Values for each version, mean differences (95% CI), medians, median differences and Intraclass Correlation Coefficients (ICCs) were calculated for all questionnaires, including relevant subscales. We chose to calculate and display both parametric and non-parametric statistics for all questionnaires as not all data met the requirements of being normally distributed and/or continuous. For the assessment of possible associations between computer skills and differences between questionnaire-versions we calculated the Spearman Correlation Coefficient. The Spearman correlation coefficient was interpreted as follows: < 0.3: none; 0.31-0.5: weak; 0.51-0.7: strong; 0.71-0.9: very strong and > 0.9: excellent. A P-value less than 0.05 (two-tailed) or a 95% confidence interval (CI) not including zero was considered statistically significant. Statistical analyses were performed using SAS version 9.1 for Windows (Chicago, IL, USA).

Rasch computer software WINSTEPS version 3.68.2 [19] was used to generate linear measures of self-reported ADL ability based on the ADL Taxonomy paper and touch-screen data Furthermore, WINSTEPS was used to analyse if any of the patients demonstrated an abnormal response pattern.

In a two-sided tests analysis for additive equivalence of paired means for a given KOOS with bounds -5 and 5 for the mean difference and a significance level of 0.05, assuming a mean difference of 0 KOOS points, a common Standard Deviation of 20, and correlation 0.95, a sample size of 20 pairs yielded a power of 0.922 (> 90%).

## Results

A total of 20 female patients with KOA were included in this trial (Figure 1), their age ranged from 54 to 76 years and with a mean of 67 years (Table 1). Approximately 90% of the patients were computer literate and comfortable using computers. With respect to their employment status and level of education, 95% were retired and 25% had only completed elementary school.

**Table 1:** Baseline characteristics of participants

Female patients with knee osteoarthritis(n = 20)		Mean; SD
Age		66.5 (7.0)
**Computer experience, education and employment**		**N (%)**
Average daily use of computers	< 5 min	3 (15)
	5-30 min	5 (25)
	> 30 min	12 (60)
Years of weekly computer use	Never	2 (10)
	< 1 year	0 (0)
	1-5 years	3 (15)
	> 5 years	15 (75)
Do you feel comfortable with using computers	No	3 (15)
	Yes	17 (85)
Working with computers is/was normal at work	No	7 (35)
	Yes	13 (65)
Education level	Elementary school	5 (25)
	High school	0 (0)
	< 4 years at University level	9 (45)
	4-6 years at University level	2 (10)
	> 6 years at University level	4 (20)
Current employment	Working full-time	0 (0)
	Working part-time	0 (0)
	Retired	19 (95)
	Unemployed	1 (5)

An overall comparison of differences between paper and touch-screen versions did not reveal any tendency towards either positive or negative values (Table 2). KOOS revealed high ICCs (0.96-0.98) and mean differences between -1.5 and 0.6. The three VAS scales displayed comparable results with ICCs between 0.88 and 0.95 and mean differences between -8.7 and 2.5 (Table 3). VAS function was significantly different between versions when applying a paired t-test, but due to lack of normal distribution of data, we also analysed this finding with a two-sided paired nonparametric test (Wilcoxon rank sum) and found no significant difference between questionnaire versions (p = 0.24). The two component summary scores in SF-36 revealed ICCs of 0.94 and 0.95 with mean differences < 0.5 (absolute). The Physical Activity Scale had an ICC of 0.93 and a mean difference of 0.4. Results from painDETECT showed a high ICC for all components (0.94-0.99). The ADL Taxonomy ability measures, revealed an ICC of 0.97. There was a mean difference of 0.5, which was tested, in a paired t-test showing a statistically significant difference between questionnaire versions (CI: 0.13; 0.95, p = 0.01). The WINSTEPS analysis revealed one patient who demonstrated an abnormal response pattern on both versions of the ADL Taxonomy. Further analysis of data from the remaining questionnaires revealed that this particular patient contributed notably to the overall variation between versions (data not shown).

**Table 2:** Scores and differences between paper and touch-screen version

	Paper (median; iqr)	Touch screen (median; iqr)	Difference (median)	Paper (mean ± SD)	Touch screen (mean ± SD)	Difference (mean, 95% CI)
KOOS; _Activities in daily living_	66.9(54.4;79.4)	65.4(51.8;82.4)	1.5	64.0 ± 22.8	63.5 ± 23.3	0.5 (-1.6;2.6)
KOOS; _Pain_	59.7(38.9;67.4)	61.1(38.9;67.4)	-1.4	55.6 ± 18.5	57.1 ± 19.3	-1.5 (-3.8;0.7)
KOOS; Q_uality of life_	46.9(35.9;56.3)	46.9(37.5;56.3)	0.0	45.6 ± 16.4	45.0 ± 14.7	0.6 (-2.4;3.6)
KOOS; _Symptoms_	60.7(38.4;71.4)	58.9(42.9;72.3)	1.8	57.9 ± 20.4	58.9 ± 20.3	-1.0 (-4.1;2.0)
KOOS; _Function in sport_	15.0(5.0;36.3)	12.5(5.0;27.5)	2.5	21.5 ± 22.8	20.5 ± 21.5	1.0 (-2.1; 4.1)
VAS _Pain_	31.5(12.8;57.3)	32.0(20.3;67.8)	-0.5	36.8 ± 25.4	40.7 ± 27.8	-3.9 (-9.4;1.6)
VAS _Function_	27.0(11.8;54.8)	34.5(25.0;69.3)	-7.5	33.2 ± 24.9	41.9 ± 25.2	-8.7 (-14.4;-3.0)
VAS _Patient global_	29.5(18.0;56.8)	24.5(12.5;58.5)	5.0	35.6 ± 22.6	33.1 ± 27.4	2.5 (-5.1;10.3)
SF-36 _Physical Functioning_	45.0(33.8;61.3)	45.0(32.5;65.0)	0.0	46.8 ± 23.3	46.8 ± 24.1	0.0 (-2.6;2.6)
SF-36 _Role Physical_	25.0(0.0;75.0)	25.0(0.0;100.0)	0.0	41.3 ± 41.6	42.5 ± 43.0	-1.2 (-15.2;12.7)
SF-36 _Bodily Pain_	42.0(41.8;44.0)	42.0(42.0;45.8)	0.0	42.1 ± 6.8	42.6 ± 7.6	-0.5 (-2.8;1.9)
SF-36 _General Health_	73.5(55.8;83.3)	69.5(51.5;87.0)	4.0	68.3 ± 21.2	68.8 ± 21.4	-0.5 (-4.1;3.2)
SF-36 _PCS_	33.6(26.4;40.5)	33.6(27.0;40.5)	-0.1	33.3 ± 7.9	33.7 ± 8.2	-0.4 (-2.1;1.4)
SF-36 _Vitality_	67.5(53.8;80.0)	75.0(55.0;80.0)	-7.5	64.0 ± 19.6	65.8 ± 22.4	-1.8 (-7.3;3.8)
SF-36 _Social Functioning_	100.0(75.0;100.0)	93.8(71.9;100.0)	6.3	85.0 ± 21.3	81.9 ± 22.4	3.1 (-0.9;6.3)
SF-36 _Role Emotional_	100.0(58.3;100.0)	83.3(33.3;100.0)	16.7	76.7 ± 36.0	70.0 ± 35.7	6.7 (-1.5;14.8)
SF-36 _Mental Health_	80.0(76.0;92.0)	84.0(80.0;92.0)	-4.0	81.6 ± 11.6	84.2 ± 9.8	-2.6 (-5.3;0.1)
SF-36 _MCS_	60.9(55.6;64.0)	58.7(52.1;65.2)	2.2	58.4 ± 8.4	58.0 ± 8.1	0.4 (-1.4;2.3)
Physical Activity Scale	44.7(37.8;52.5)	44.3(37.5;52.5)	0.4	45.3 ± 9.4	44.9 ± 9.6	0.4 (-2.0;2.7)
painDETECT _Strength of pain_	3.0(1.8;6.3)	3.0(2.0;6.3)	0.0	3.8 ± 2.5	3.9 ± 2.3	-0.1 (-0.4;0.1)
painDETECT _Strongest pain_	5.0(3.8;7.3)	5.5(3.8;7.3)	-0.5	5.2 ± 2.7	5.5 ± 2.2	-0.3 (-0.7;0.1)
painDETECT _Average pain_	4.0(3.0;7.0)	4.0(3.0;7.0)	0.0	4.6 ± 2.3	4.9 ± 2.1	-0.3 (-0.8;0.1)
painDETECT _Pattern of pain_	2.0(1.0;3.0)	2.0(1.0;2.3)	0.0	2.0 ± 0.9	1.9 ± 0.9	0.1 (-0.1;0.3)
painDETECT _Radiation of pain_	1.0(0.0;1.0)	1.0(0.8;1.0)	0.0	0.7 ± 0.5	0.8 ± 0.4	-0.1 (-0.2;0.1)
painDETECT _Pain quality_	1.1(0.5;1.8)	1.2(0.8;1.8)	-0.1	1.3 ± 0.9	1.3 ± 0.9	0.0 (-0.2;0.1)
painDETECT _Score_	8.5(5.0;14.0)	9.0(6.0;12.3)	-0.5	10.3 ± 7.0	10.3 ± 7.1	0.0 (-0.95;0.95)
ADL Taxonomia	4.4(2.4;7.3)	3.7(1.7;6.3)	0.8	4.3 ± 2.6	3.8 ± 2.7	0.5 (0.13;0.95)

**Table 3:** Intraclass Correlation Coefficients between paper and touch-screen versions

HSQs	Intraclass Correlation Coefficient (ICC)
KOOS; _Activities in daily living_	0.98
KOOS; _Pain_	0.98
KOOS; _Quality of life_	0.96
KOOS; _Symptoms_	0.97
KOOS; _Function in sport_	0.98
VAS _Pain_	0.95
VAS _Function_	0.91
VAS _Patient global_	0.88
SF-36 _Physical Functioning_	0.99
SF-36 _Role Physical_	0.86
SF-36 _Bodily Pain_	0.87
SF-36 _General Health_	0.97
SF-36 _PCS_	0.95
SF-36 _Vitality_	0.91
SF-36 _Social Functioning_	0.97
SF-36 _Role Emotional_	0.93
SF-36 _Mental Health_	0.91
SF-36 _MCS_	0.94
Physical Activity Scale	0.93
painDETECT _Strength of pain_	0.99
painDETECT _Strongest pain_	0.97
painDETECT _Average pain_	0.95
painDETECT _Pattern of pain_	0.94
painDETECT _Radiation of pain_	0.94
painDETECT _Pain quality_	0.98
painDETECT _Score_	0.98
ADL Taxonomia	0.97

Computer skills, age and/or education did not have any impact on differences between questionnaire-versions, we found an unsystematic pattern of non-significant correlations ranging from 0.05 to 0.40 (p-values > 0.05).

**Figure 2:**
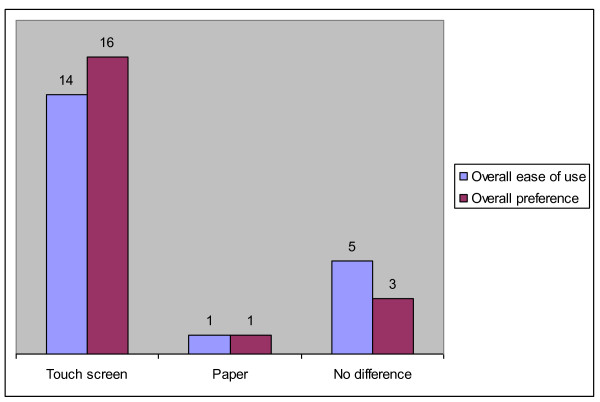
**Patients' preferences for either touch screen or paper versions of HSQs in general**.

When patients answered questions addressing their overall satisfaction with this new questionnaire-modality, we found that 16 out of 20 patients preferred touch screen questionnaires over paper versions. Furthermore, only one patient preferred the paper to the touch-screen version (Figure 2). Among patients who expressed a preference, significantly more stated that the touch screen version was easier and generally preferable.

Time spend on answering questionnaires on touch screen was measured, and results revealed that patients on average spend 6 minutes on SF-36, 0.5 minutes on the VAS scales from the OMERACT-OARSI responder criteria, 2.2 minutes on painDETECT, 9 minutes on the ADL taxonomy and 7 minutes on KOOS.

Also, despite the presence of an instructor during the trial, no patients needed any tutoring related to the touch screen versions of the questionnaires.

### Missing data

In this study we experienced no missing data due to the complete real-time saving of computer data and due to the fact that, clinical staff manually checked all paper versions before patients were allowed to leave the session.

## Discussion

Our aim was to validate touch-screen self-assessment questionnaires for use in the clinic. Comparing paper and touch-screen versions of our selected questionnaires, which covered what is normally used in a Rheumatology setting, our overall finding was a very high agreement between PROs obtained via the paper and the touch-screen versions.

Our results revealed that retired elderly female patients do not experience any problems when using computerized questionnaires, which implies that this method is applicable for the majority of patients in the clinical Rheumatology setting.

Results from our study of KOOS, VAS measurements, and SF-36 are comparable to test-retest results reported in earlier studies [20-24]. While several studies have compared the Physical Activity Scale questionnaire to accelerometers and pedometers [25], test-retest reliability has not yet been evaluated. Consequently our results may only be compared to other questionnaires assessing METs, which in one case found similar results in patients with hip and/or knee osteoarthritis [26]. The original validation of painDETECT did not include a test-retest evaluation as the authors believed that symptoms of pain would fluctuate so much that such a test would only have limited use [15]. We, therefore, present the first results regarding such a test, and overcome their consideration on fluctuation by having a short period of time between tests. The ADL taxonomy showed that all patients had similar or higher scores on paper compared to touch screen (data not shown) with a mean difference of 0.5 (CI: 0.13; 0.95). Viewing data on a Bland-Altman plot showed that with higher mean values, differences go toward zero. This overall difference may be due the fact that questions addressing easy tasks are presented at the beginning of the questionnaire, and that patients tend to continue the answering of subsequently more difficult questions at the same level when viewing all questions simultaneously in the paper version. A similar observation was done in a previous analysis of differences between questionnaire- and interview-based measures of ADL ability [18]. The touch screen version presents a single question at a time, and may imitate an interview setting that force the patient to a more active consideration of each answer.

Our analyses of the 10-point-Likert scales from painDETECT and the three VAS scores suggest differences in the test-retest results (Table 2 and 3). Direct comparison shows that test-retest ICCs increase and difference diminishes when applying 10-point-Likert scales instead of 100 mm VAS'; a finding that might have implications for future research strategies.

An unforeseen bonus was the patient's positive attitude towards touch-screens. Touch-screen questionnaires were rated preferential and easier to paper versions, independent of level of computer use and skills.

The lack of correlation between previous computer experience and differences between questionnaires has also been reported in other studies, where use of touch-screen questionnaires was reported less stressful and requiring less or no help from staff to understand how to use them [3]. This may be due to only getting one question at the time, and thereby avoiding problems created by interruptions [27].

Based on our present study, we conclude that our newly developed computer-assisted touch-screen questionnaires for PROs are directly comparable and therefore valid for recording of these data in the clinic as well as in research studies. This is in agreement with other studies comparing paper versions with touch screen for the bath AS questionnaires and the Quebec Scale [16] for the QOLRAD questionnaire [28], for WOMAC 3.1 [6], for RAQol, HAQ and VAS [29], for short-form McGill and Pain disability Index [30], for HAQ [31], for quality of life questionnaires [32], and for quality of care questionnaire [33].

Limitations for this study were that most participants were computer literate, and we can therefore not conclude whether or not all patients can use this kind of computer technology. Even so, we know that touch screens are used daily in the collection of data for the DANBIO database; gathering patient reported outcomes from the majority of rheumatologic patients in Denmark [31,34].

In order to examine the test-retest of the chosen questionnaires, we had to consider several things; patients were not to be excessively tired, the test-retest was to consider possible fluctuation of symptoms and the setup should be so that most patients would accept participation. The chosen setup was a 5-minute interval between versions and questionnaires, as we believed that this offered a reasonable total time use (on average, 60 minutes for answering all questionnaires plus 60 minutes for pauses). Also, for several reasons, we did not believe that recall bias was a major issue in our setup. Many questionnaires were long and time-consuming, the answering of 12 questionnaires does not allow people to memorize a significant part of given answers and the study design (randomization) should level out any significant bias arisen by patients starting with a specific questionnaire. Also, we only included females in this trial, and precautions should be taken when extrapolating results to males; even so, we do not believe that significant differences between genders are to be expected.

With our broad and extended collection of questionnaires, the touch-screens open for further development towards more frequent self-assessment. Another potential of this system is the possibility of transferring answers from self assessment forms to other health institutions, e.g. from a hospital clinic to the GP or to another specialist, and in the electronic form, it will be part of the electronic patient notes in its original form. Data completeness is assured in our software version, as all items must be answered before continuation. The last advantage is the clear marked improvement of data by abolishing key-in errors, as well as the elimination of costs related to entering paper-based data into databases and the manual double-checking of data. As a future perspective, the patients will be able to answer questionnaires from home and may avoid some of the check-up visits, which are a burden of chronic patients.

## Conclusion

The overall aim of this study was to investigate the prospect of introducing computerized questionnaires for patients with KOA. The study showed that touch-screen self-assessment questionnaires in the Rheumatology clinic are as reliable as paper questionnaires. The only observed difference, between the two versions of the ADL taxonomy, could be partly accounted for. The patients in general prefer touch screen and further advantages are less need for staff assistance, no errors related to processing of paper versions, and elimination of missing and/or incomplete data.

## Competing interests

The authors declare that they have no competing interests.

## Authors' contributions

HG drafted the study design, protocol, analysis and interpretation of data as well as the manuscript. The technical solution was developed by PK, who also contributed to the interpretation of data and revised the manuscript. Key persons in the statistical analyses were EW and RC and they also contributed to the design, interpretation of data and revisions of the manuscript. EMB, BDS and HB contributed to the overall design idea, protocol descriptions and revisions the manuscript. All authors approved the final version.

## Pre-publication history

The pre-publication history for this paper can be accessed here:

http://www.biomedcentral.com/1471-2474/12/190/prepub
